# Sarcomere Lengths Become More Uniform Over Time in Intact Muscle-Tendon Unit During Isometric Contractions

**DOI:** 10.3389/fphys.2020.00448

**Published:** 2020-05-12

**Authors:** Eng Kuan Moo, Walter Herzog

**Affiliations:** Human Performance Laboratory, Faculty of Kinesiology, University of Calgary, Calgary, AB, Canada

**Keywords:** sarcomere contraction dynamics, sarcomere length non-uniformity, extracellular matrix, sliding filament theory, second harmonic generation imaging, skeletal muscle properties

## Abstract

The seemingly uniform striation pattern of skeletal muscles, quantified in terms of sarcomere lengths (SLs), is inherently non-uniform across all hierarchical levels. The SL non-uniformity theory has been used to explain the force creep in isometric contractions, force depression following shortening of activated muscle, and residual force enhancement following lengthening of activated muscle. Our understanding of sarcomere contraction dynamics has been derived primarily from *in vitro* experiments using regular bright-field light microscopy or laser diffraction techniques to measure striation/diffraction patterns in isolated muscle fibers or myofibrils. However, the collagenous extracellular matrices present around the muscle fibers, as well as the complex architecture in the whole muscles may lead to different contraction dynamics of sarcomeres than seen in the *in vitro* studies. Here, we used multi-photon excitation microscopy to visualize *in situ* individual sarcomeres in intact muscle tendon units (MTUs) of mouse tibialis anterior (TA), and quantified the temporal changes of SL distribution as a function of SLs in relaxed and maximally activated muscles for quasi-steady state, fixed-end isometric conditions. The corresponding muscle forces were simultaneously measured using a force transducer. We found that SL non-uniformity, quantified by the coefficient of variation (CV) of SLs, decreased at a rate of 1.9–3.1%/s in the activated muscles, but remained constant in the relaxed muscles. The force loss during the quasi-steady state likely did not play a role in the decrease of SL non-uniformity, as similar force losses were found in the activated and relaxed muscles, but the CV of SLs in the relaxed muscles underwent negligible change over time. We conclude that sarcomeres in the mid-belly of maximally contracting whole muscles constantly re-organize their lengths into a more uniform pattern over time. The molecular mechanisms accounting for SL non-uniformity appear to differ in active and passive muscles, and need further elucidation, as do the functional implications of the SL non-uniformity.

## Introduction

The striation patterns of skeletal muscle carry important functional information, as they reflect the amount of overlap between the thick and thin filaments within a sarcomere, and hence the amount of steady-state, maximal, isometric force that a muscle can generate (Gordon et al., 1966). The seemingly uniform striation spacing, however, has been shown to be inherently non-uniform across all hierarchical levels: the intact muscle (Llewellyn et al., 2008; Cromie et al., 2013; Moo et al., 2016; Lichtwark et al., 2018), isolated muscle fibers or myofibrils (Huxley and Peachey, 1961; Joumaa et al., 2008; Pavlov et al., 2009; Infantolino et al., 2010; Johnston et al., 2016). The non-uniform striation pattern, also known as sarcomere length (SL) non-uniformity/dispersion, has been quantified as the extent of deviation of individual sarcomere lengths (SLs) from the average value of a group of sarcomeres. The SL non-uniformity seen in a relaxed muscle has been found to increase when a muscle is activated (Julian and Morgan, 1979; Joumaa et al., 2008; Sanchez et al., 2015; Moo et al., 2017a; Moo and Herzog, 2018). This activation-induced increase of SL non-uniformity has been theorized to play an important role in a series of functional properties of skeletal muscle, such as force creep in isometric contractions (Gordon et al., 1966; Edman and Reggiani, 1984), force depression following shortening of activated muscle (Morgan et al., 2000), and residual force enhancement following lengthening of activated muscle (Morgan, 1990; Edman and Tsuchiya, 1996).

To date, our knowledge of sarcomere contraction dynamics has been derived primarily from experiments using isolated muscle fiber or myofibrils where the striation (Huxley and Peachey, 1961; Telley et al., 2006; Infantolino et al., 2010) and diffraction (ter Keurs et al., 1978; O’Connor et al., 2016; Moo et al., 2017b) patterns can be captured using bright-field microscopy and laser diffraction techniques, respectively. Due to the technical difficulties associated with observing individual sarcomeres in a whole muscle, there is a lack of understanding of the dynamic behavior of sarcomeres residing in muscles, where thousands of muscle fibers are assembled by collagenous extracellular matrices in a complex architecture (Purslow, 1989, 2008; Heemskerk et al., 2005; Azizi et al., 2008; Lovering et al., 2013). The extracellular matrices provide structural support to the muscle, the muscle fibers and myofibrils (Purslow, 1989, 2008; Gillies and Lieber, 2011) and could potentially lead to a different contraction dynamics than the sarcomeres in isolated myofibrils or muscle fibers that only have part or none of the extracellular matrix of the muscle. As the muscle and its sarcomeres elongate over the descending limb of the force-length curve, the actomyosin-based active forces decrease (Gordon et al., 1966), whereas the passive forces caused by the extracellular matrices and intracellular titin increase (Gordon et al., 1966; ter Keurs et al., 1978; Leonard and Herzog, 2010; Wood et al., 2014). This shift in relative force contribution between passive structural and active contractile elements may potentially alter the sarcomere contraction dynamics, thereby affecting force production. In view of the apparent importance of SL non-uniformity on muscle properties, tracking the time history of SL distribution in whole muscles prior to and during muscle activation may provide new insight into the influence of extracellular matrices on sarcomere mechanics and inter-sarcomeric interactions.

Therefore, the purpose of the current study was to systematically quantify the temporal changes of SL distribution as a function of SLs in quasi-steady state of relaxed and maximally activated muscles. The MTU of TA of living mice was stretched to five lengths, after which the TA was either left unstimulated (for the relaxed condition), or maximally activated by electrical stimulation of the sciatic nerve (for the activated condition). The *in situ* sarcomeres from a small region at mid-belly of the TA were visualized by multi-photon imaging and the corresponding muscle forces were measured by a force transducer. The SL distribution was investigated by quantifying the dispersion of SLs through the coefficient of variation (CV). We hypothesized that the time-varying SL dispersion depended on the initial SLs, and differed between relaxed and activated muscles.

## Materials and Methods

The analysis of the current study was based on a data set previously published for a different purpose and using different analysis procedures (Moo et al., 2020).

### Animal Preparation, Imaging of Sarcomeres, and Measurement of Muscle Forces

All aspects of animal care and experimental procedures were carried out in accordance with the guidelines of the Canadian Council on Animal Care and were approved by the University of Calgary’s Life Sciences Animal Research and Ethics Committee. All animals were euthanized at the end of the experiment. The experimental procedures were the same as described in Moo et al. (2020). Briefly, 10–12 week-old male C57/BL6 mice (*N* = 6; 30.5 ± 2.6 g) were anesthetized using a 1–2% isoflurane/oxygen mixture. A cuff-type bipolar electrode was implanted on the sciatic nerve. The left proximal femur was fixed using a custom-made clamp at a knee flexion angle of 60°. The tibialis anterior (TA) muscle was surgically dissected from the tibia and surrounding muscles. The proximal end of the TA remained attached to it’s *in situ* attachment site on the proximal tibia; whereas the distal, tendon end of the TA was detached from its insertion site with a remnant piece of the first metatarsal bone. The distal end was then firmly attached to a tendon clamp that extended from a 3-axes, linear micro-manipulator (Newport Corp., CA, United States) which, in turn, was instrumented with a uniaxial force transducer (LCFL-1 kg, Omega Engineering Inc., CT, United States) and a rotary stepper motor (PG25L-D24-HHC1, NMB Technologies Corporation, MI, United States) for measurement of muscle force and computer-controlled stretching of the TA at a constant speed, respectively. A piece of parafilm (VWR, Canada) was stitched to the skin overlying the TA and attached to the tendon clamp to form a pool to accommodate a phosphate buffered saline solution that kept the muscle hydrated and allowed for imaging using a water-immersion objective.

Sarcomere images were captured, and muscle forces were measured, in relaxed and activated muscles at five MTU lengths. A fluorescent microsphere-based marker (Thermo Fisher Scientific Inc., MA, United States) was attached to the mid- region of the muscle using a 100 μm-diameter glass pipette tip to allow for the tracking of the same muscle volume in the relaxed and activated muscles. This tracking of the muscle volume was particularly important as the muscle fibers move proximally during activation, and the magnitude of this movement depends critically on the MTU. Sarcomeres were visualized by second harmonic generation (SHG) imaging of the TA using an upright, multi-photon excitation microscope (FVMPE-RS model, Olympus, Tokyo, Japan) equipped with an ultrafast laser (InSight DeepSee-OL, Spectra-Physics, CA, United States) and a 25x/1.05NA water immersion objective (model XLPLN25XWMP2-Ultra, Olympus, Tokyo, Japan). The laser wavelength was 800 nm. The SHG signals emitted by sarcomere A-bands were collected in the backward (epi-) direction through the objective lens using a band-pass filter at the harmonic frequency (FF01 400/40, Semrock Inc., NY, United States). The average laser power in the sample plane was kept between 15 and 18 mW to avoid thermal damage to the muscle and to ensure optimal images. Time-series, two-dimensional image bands were acquired at a frame rate of 23 frames/s in the horizontal plane (dimension: 159 × 2.8 μm; pixel size: 0.2 μm; bit-depth: 12; dwell time: 2 μs) from an imaging area of 159 × 159 μm at the top 50 μm of the TA.

In each trial, the TA was first held at a resting reference length (11–12.5 mm) before being stretched by 0.5, 1.5, 2.0, 3.0, or 4.0 mm at a speed of 0.5 mm/s. When the target MTU length was reached, the TA was activated using a 600 ms, continuous, supra-maximal electrical stimulation [3 ×α-motor neuron threshold, 70–80 Hz, 0.1 ms square wave pulse (Grass S88, Astro-Med. Inc., RI, United States)] of the sciatic nerve, and sarcomere imaging was performed simultaneously. In the relaxed muscle, sarcomere imaging was performed without activation. Following the sarcomere imaging and force measurement, the TA was returned to its resting reference length and a 2 min rest period was given between trials. Muscle forces were recorded by a data acquisition unit (DI-149, DATAQ Instruments, Inc., OH, United States) at 500 Hz throughout all trials.

### Analysis of Sarcomere Images and Muscle Forces

The experimental trials were initially divided into ten groups based on the two muscle states (relaxed vs. activated) and the five MTU lengths. Four to six trials with good sarcomere images (i.e., good signal-to-noise ratio and minimal motion artifact) and muscle forces were selected from each group for final analysis.

**FIGURE 1:**
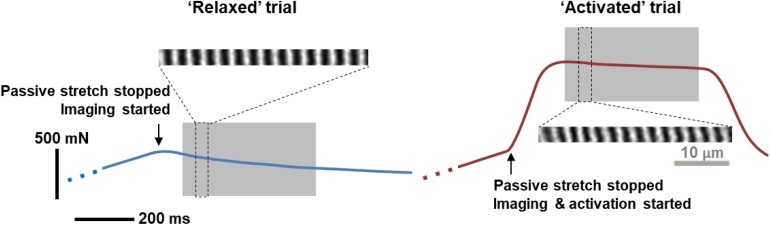
A representative example of force traces recorded, and the corresponding sarcomere images, when the MTU was stretched by 4 mm and held at this length for either passive stress relaxation (left, blue curve) or for a fixed-end isometric contraction (right, red curve). Sarcomere images taken during the gray-shaded, quasi-steady state, isometric contractions were analyzed for time dependence of SL distribution. During this period, there was a time-dependent decrease in force due to stress relaxation in the relaxed muscles, and due to muscle fatigue in activated muscles, respectively. Note that the sarcomere lengths were measured as the distance between the centroids of adjacent A-bands (bright bands in the figure), rather than following the typical definition of sarcomere length between two adjacent Z-lines.

The 2.8 μm-wide planar image bands typically contained two parallel-running myofibrils (Powers et al., 2016) and 5–30 sarcomeres in series. Sarcomeres were visible between 200 and 600 ms of the contraction after maximal isometric forces were developed in the activated muscles (Figure 1, quasi-steady state marked by the region shaded in gray). The selected image bands were band-pass-filtered using Fiji software (National Institutes of Health, MD, United States), and were processed using a custom-written MATLAB code that identified the intensity-weighted centroids of the sarcomeric A-bands. Individual SLs were measured as the distance between adjacent A-band centroids (Yang et al., 1998; Cromie et al., 2013; Moo et al., 2016). The surface tilt of the local muscle region was measured using a through-thickness muscle image, and SLs were corrected for out-of-plane orientation (Moo et al., 2017a; Moo and Herzog, 2018). The morphometric measures that were derived from individual image bands include the mean, standard deviation (SD), CV (= SD/mean), 95th percentile and 5th percentile of the SLs, as well as the sarcomere length range, which is defined as the difference between the 95th and the 5th percentiles of the SLs. Details of the numbers of analyzed images and sarcomeres can be found in Supplementary Table S1.

The force values measured from the uniaxial force transducer represented a combination of passive and active forces, which are the forces resulting from the passive structural elements and the contractile components of the muscles, respectively. A structural Hill-type model was used to extract passive and active forces from the total measured force (MacIntosh and MacNaughton, 2005; Rode et al., 2009; Moo et al., 2020). Briefly, using the mean SLs measured in the activated muscles, the passive force was determined from the passive force-length curve, which was constructed by best-fitting an exponential curve to the force-SL data of the relaxed muscles in individual animals. The active force could then be calculated by subtracting the passive force from the total measured force. A representative example of a sarcomere FL curve measured in the relaxed or activated conditions in a single animal was included in Supplementary Figure S1.

The force-time history of each trial was divided into segments of equal intervals that correspond to the acquisition time of individual sarcomere image bands (43 ms). The average force within each time-segment was calculated and linked temporally to the morphometric measures derived from the sarcomere image that was captured within the same time interval. The amount of force loss from the maximal force recorded in the isometric period (Figure 1) of the relaxed and activated muscles was also calculated for each time segment.

### Group Re-assignment for Analysis of Temporal Changes of Sarcomere Length Distribution

**FIGURE 2:**
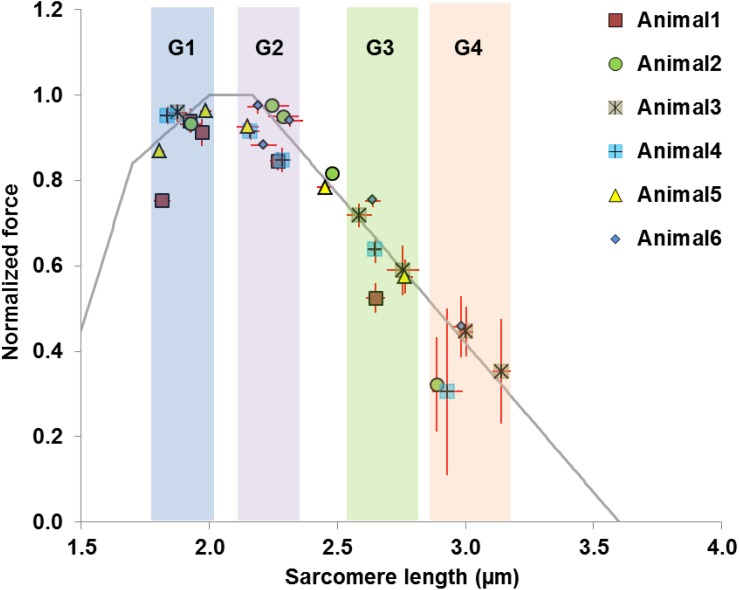
Definition of data groups used in the current study. Rather than using the MTU length as the grouping parameter, the data were divided into four groups (G1–G4) based on the average SLs measured in the mid-belly of the activated muscles. The theoretical sarcomere force-length curve (gray) was overlaid for reference, with the optimal SLs ranging from 2.0 to 2.17 μm (Walker and Schrodt, 1974; Herzog et al., 1992; Gokhin et al., 2014, 2015; Moo et al., 2020). Each group of sarcomere lengths occupies different regions of the force-length curve, and contains sarcomeres that differ by 0.15–0.25 μm. Group 1 consists of sarcomeres on the ascending limb (1.8–2.0 μm). Group 2 contains sarcomeres residing on the plateau or at the start of the descending limb, with lengths ranging from 2.15 to 2.30 μm. Group 3 contains sarcomeres at lengths between 2.55 and 2.75 μm where passive forces become substantial (Wood et al., 2014; Moo et al., 2020). Group 4 is composed of sarcomeres that are located further down the descending limb with lengths ranging from 2.90 to 3.15 μm. Note that two data points from Animal 2 and Animal 5 were excluded as they fell outside the grouping criteria.

**FIGURE 3:**
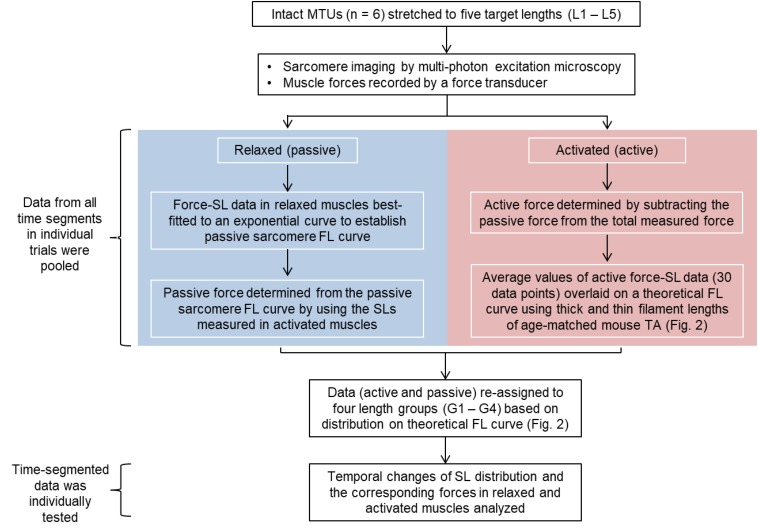
Flow chart summarizing the work flow of data acquisition and processing. Sarcomere lengths were measured as the distance between two adjacent A-band centroids. The muscle force-time data were divided into equal time (43 ms) segments that correspond to the frame rate of the multi-photon excitation microscopy. The morphometric data were then temporally linked to the time-segmented muscle force in individual trials (see Materials and Methods for better description).

The average values of the morphometric and force data were, respectively, determined from the image bands and force transducer readings of all time segments collected at the five MTU lengths of the individual muscles for the relaxed and activated conditions. The active sarcomere force-length curves were plotted for the six tested animals (Figure 2). Based on the average active SLs determined at five MTU lengths, the data were re-assigned into four SL groups (G1–4) that span across the ascending limb, plateau, and descending limb of the theoretical force-length curve (Figure 2), built based on the thick and thin filament lengths of age-matched mice (Walker and Schrodt, 1974; Herzog et al., 1992; Gokhin et al., 2014, 2015; Moo et al., 2020). Each SL group contained sarcomeres with average lengths that differed by 0.15–0.25 μm (see Supplementary Table S2). The temporal changes of SL distribution and the corresponding forces were analyzed based on this new grouping definition. The work flow of data acquisition and processing is summarized in Figure 3. Note that the reproducibility of SL distribution between trials was not investigated here due to the experimental design that did not allow for such an analysis. Interested readers can refer to the Supplementary Material where we address this issue in more detail and provide some conceptual results of pilot work on single myofibrils.

### Statistical Analysis

Statistical analyses were performed using SPSS (version 25, SPSS Inc., IL, United States). Unless otherwise stated, the results were expressed as estimated marginal means (EMM) ± standard error (S.E.). The data from all time segments in individual trials were first pooled, and the mean values of the mean, CV of SLs, as well as the muscle forces were analyzed for condition effects, which include muscle states (relaxed vs. activated) and SL groups (G1–4), using a generalized estimating equation (GEE, under Genlin Mixed procedures in SPSS) to take into account the correlated (measurements within individual animals) nature of the observations and the unbalanced (unequal number of serial measurements for each animal) design of the study. Then, the time-segmented data in these trials were analyzed by GEE to individually test for relationships between contraction time and the mean SL, CV, 5th percentile, 95th percentile, and length range of SLs, as well as force drop during the isometric period. All statistical tests were performed two-sided with type I error, α, set at 0.05 level. Multiple comparisons were accounted for through sequential Sidak adjusted *p*-values. As long as the data were interval-type, no assumptions regarding normal distribution was needed for the GEE statistical method.

## Results

**FIGURE 4:**
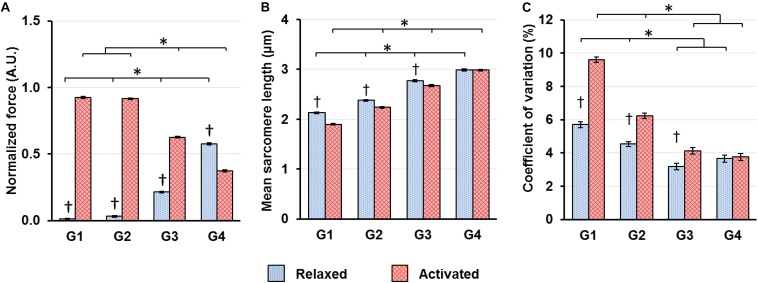
**(A)** Muscle forces, **(B)** mean SLs and **(C)** coefficient of variations of SLs, which are a measure of SL non-uniformity, in the relaxed (blue bars) and activated (red bars) muscles averaged over the isometric period indicated in Figure 1. **(A)** Muscle forces were normalized by the highest isometric force recorded (2570 ± 128 mN, mean ± S.E.) in individual animals. Active forces decreased whereas passive forces increased as SLs increased from G1 to G4. In G1–G3, the active forces were substantially higher than the passive forces. In G4, the passive forces became higher than the active forces; **(B)** Activation led to shortening of *in situ* sarcomeres in a SL-dependent manner. The magnitude of sarcomere shortening decreased from 10.8 to 3.5% while going from G1 to G3. In G4, there was no sarcomere shortening associated with activation; **(C)** Activation led to a SL-dependent increase of CVs of SLs, with the magnitude becoming smaller as sarcomeres lengthened from G1 to G3, and becoming virtually zero in G4. ^∗^Indicates significant differences between SL groups for either the relaxed or the activated condition (*p* < 0.01). ^†^Indicates significant differences between the relaxed and activated states within a SL group (*p* < 0.01).

When the sarcomeres were lengthened from G1 to G4, the relative contribution of passive and active forces to the total forces varied. The passive forces were negligible in G1–G2, but grew to take up 25.4% of the total forces in G3. In G4, 60.6% of the total forces were contributed by the passive forces, while the remaining 39.4% came from the active forces (Figure 4A). Sarcomeres on the ascending limb of the theoretical force-length curve (G1) shortened by 10.8% upon muscle activation. But the magnitude of this activation-induced shortening became smaller and was 5.9 and 3.5% for the G2 and G3 length conditions, respectively (Figure 4B). For the G4 length condition, activation did not result in any sarcomere shortening (Figure 4B). As to the SL dispersion, the CV of SLs for the G1 length condition increased by 68.3% upon muscle activation (Figure 4C). This activation-induced increase of CV reduced to 37.6 and 29.6% for the G2 and G3 length conditions, respectively, and became negligible for the G4 length condition (Figure 4C).

**FIGURE 5:**
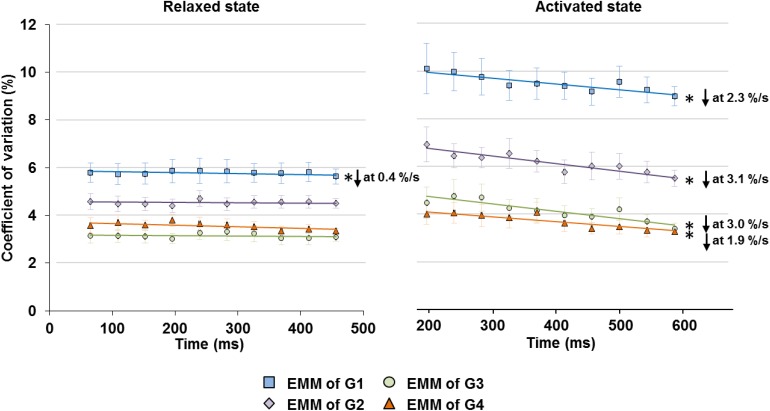
Changes in SL non-uniformity, quantified in CV of SLs, in the relaxed (left graph) and activated (right graph) muscles over the isometric, quasi-steady state indicated in Figure 1. In the relaxed state, only sarcomeres in G1 showed a slight decrease in CV at a rate of 0.4%/s, while the rest of the groups did not show any linear relationship between CV and time. In the activated state, sarcomeres in G1–G4 all experienced a decrease of CV over the 400 ms isometric activation at a rate of 1.9–3.1%/s. ^∗^Indicates significant relationship between CV and time (*p* < 0.01). EMM, estimated marginal mean.

When the relaxed muscles were held at fixed lengths, the CV of SLs on the ascending limb (G1) decreased slightly at a rate of 0.4%/s, but the CVs in G2–G4 did not show any time-dependence (Figure 5 left panel). Average SLs and sarcomere length range also showed negligible or no change with time (Supplementary Figure S2).

**FIGURE 6:**
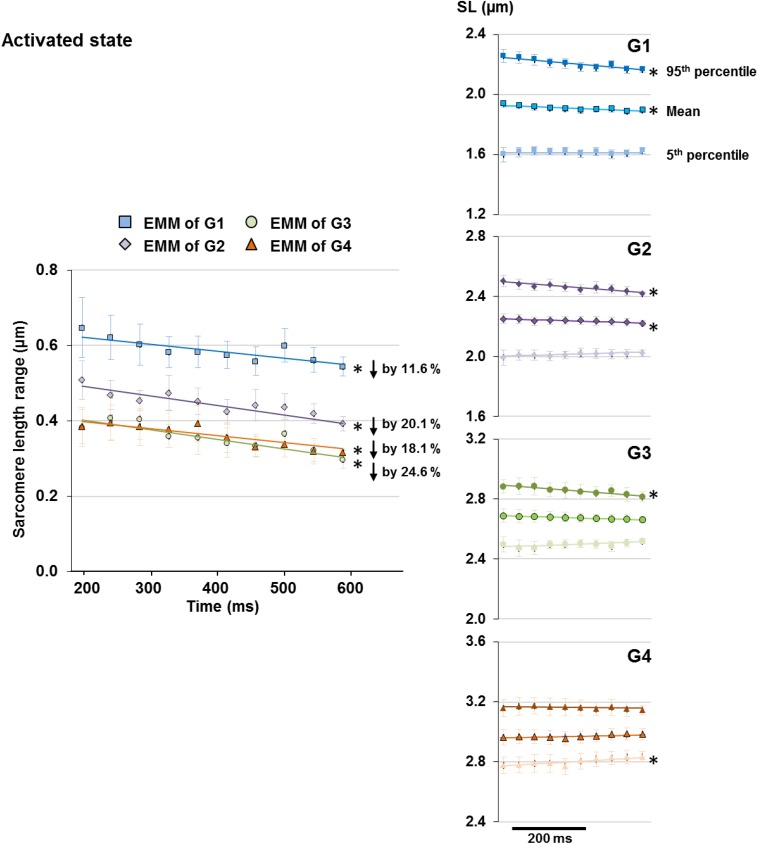
Length range of sarcomeres (difference between 95th and 5th percentiles of SLs) observed in individual image bands of activated muscles became 11.6 and 24.6% narrower following the 400 ms quasi-steady state of isometric contractions at all length conditions (G1–4, left column). However, the manner by which the sarcomere length range decreased differed between groups. Plots of the changes of average, 5th percentile, and 95th percentile of the SLs with time (right column) revealed that over the time of fixed-end isometric contraction, the shortest (5th percentile) sarcomeres remained at constant lengths but the longest (95th percentile) sarcomeres shortened by 2.5–3.6% for G1–G3; whereas the opposite occurred in G4 in which the shortest sarcomeres were elongated by 1.9% while the longest sarcomeres stayed at constant lengths. Note that the decrease in length range in G1 and G2 was accompanied by a slight 1.2–1.9% decrease of average SLs over the 400 ms. ^∗^Indicates significant relationship between time and either SL range, 5th percentile, average, or 95th percentile of the SL (*p* < 0.01). EMM, estimated marginal mean.

Upon isometric activation, SL dispersion increased instantaneously, but decreased at subsequent time points at a rate of 1.9–3.1%/s for all length conditions (G1–G4), which was 5–8 times faster than that in sarcomeres of relaxed muscles (Figure 5, right panel). During the 400 ms quasi- steady state of activation, the average SLs decreased slightly by 1.2–1.9% in G1 and G2, but remained constant in G3 and G4 (Figure 6 right column); whereas sarcomere length range became 11.6–24.6% narrower (G1–G4, Figure 6 left column). When the shortest (5th percentile) and longest (95th percentile) sarcomeres in individual image bands were separately analyzed, it was found that for the G1–G3 length conditions, the shortest sarcomeres remained at constant lengths whereas the longest sarcomeres became 2.5 and 3.6% shorter over time (Figure 6, right column). The opposite behavior was found for the G4 length condition, in which the shortest sarcomeres were elongated by 1.9% but the longest sarcomeres stayed at a constant length at the end of the isometric contraction (Figure 6, right column).

**FIGURE 7:**
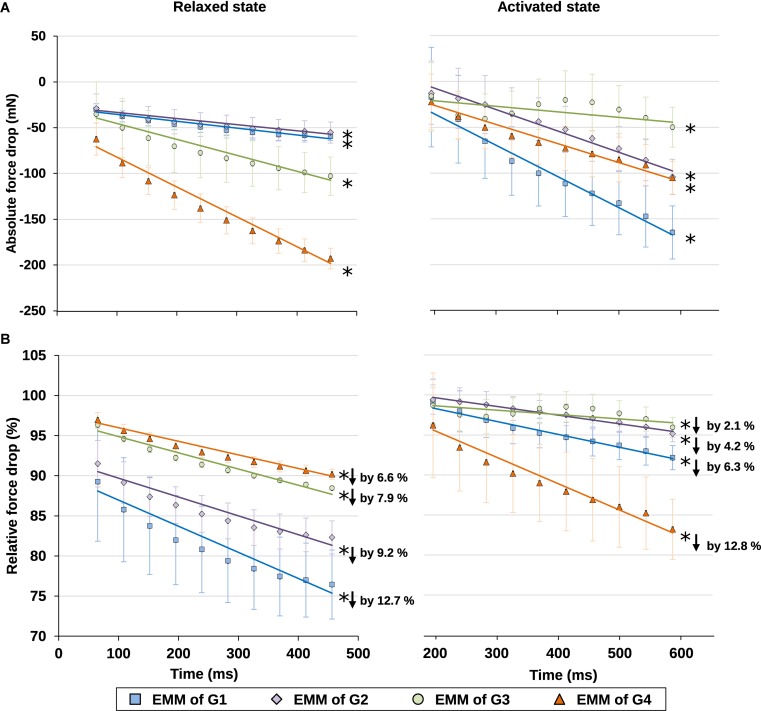
**(A)** Absolute values and **(B)** relative values of force drop from the maximal isometric force increased with time in isometrically held, relaxed (left) and activated (right) muscles. For the relative force drops, the forces were compared, respectively, to the maximal passive (left) or active (right) forces in individual trials (see Supplementary Table S3 for more details on the active and passive forces used for normalization). The force dropped, respectively, by 6.6–12.7% due to stress relaxation in the relaxed muscle (**B**, left graph), and by 2.1–12.8% due to muscle fatigue in the activated muscle (**B**, right graph) during the 400 ms isometric period. ^∗^Indicates significant relationship between force drop and time (*p* < 0.01). EMM, estimated marginal mean.

Finally, the time-dependent decreases in force were also analyzed for the relaxed and activated muscles. The force losses were expressed in absolute and relative values (Figure 7). For the relative force losses, the forces were normalized by the maximal passive or the maximal active forces for the individual passive and active trials, respectively. It was found that the magnitude of force loss was similar for the relaxed and activated muscles. Forces decreased by 6.6–12.7% in the relaxed, and by 2.1–12.8% in the activated muscle (Figure 7B).

## Discussion

Here, we investigated the time-dependent changes of SL non-uniformity as a function of SLs in the relaxed and activated muscles, and also probed into the roles of relative force contribution from the passive structural and active contractile components on the contraction dynamics of the sarcomeres in intact MTUs. Consistent with the literature (Llewellyn et al., 2008; Cromie et al., 2013; Moo et al., 2016; Lichtwark et al., 2018), our results indicate that SL distribution in the relaxed muscles was inherently non-uniform (Figure 4C). Upon supra-maximal, isometric activation of the muscles, SLs were perturbed into a more non-uniform state, with muscles/sarcomeres at the short lengths (G1–G2) experiencing greater changes than muscles/sarcomeres at the long lengths (G3–G4, Figure 4C). However, regardless of the level of SL non-uniformity in the first 150–200 ms of activation, sarcomeres tended to re-organize themselves into a more uniform length distribution over time, even at lengths which were once thought to be the “unstable” descending limb of the force-length relationship. Contrary to the “instability” theory proposed by Hill (1953), Gordon et al. (1966) and Morgan (1990, 1994), SLs on the descending limb were not diverging, and sarcomeres were not rapidly pulled beyond actomyosin overlap. Rather, SL non-uniformity, quantified by the CV, decreased at a rate of 1.9–3.1%/s in all SL groups (G1–G4, Figure 5) of the activated muscles, with minimal or no change in average SLs (Figure 6). The force drop during the quasi-steady state (Figure 1) was unlikely to contribute to the decrease of SL non-uniformity, as force drops were found in both the activated and relaxed muscles, but the CV of SLs in the relaxed muscles either decreased slightly or remained constant with time (Figure 5). Considering that the highest CV observed here was ∼10%, a decrease of CV of 3.1% per second was substantial. Similar findings were also reported in previous studies (Moo et al., 2017a; Moo and Herzog, 2018).

As the decrease in SL non-uniformity occurred exclusively in the activated muscles, the active force-producing mechanism likely plays a role in this re-distribution of SLs. During muscle activation, sarcomeres generate force through cross-bridge attachments to actin, which are influenced by the calcium-mediated activation of actin (thin) filaments (Gordon et al., 2000), and the force-mediated activation of mechano-sensing myosin (thick) filaments (Linari et al., 2015; Fusi et al., 2016). The cross-bridges of the thick filaments respond to increased force by undergoing conformational changes from the “super-relaxed” to the “on” state in a positive feedback loop, thereby increasing the proportion of active cross-bridges for force production (Linari et al., 2015; Fusi et al., 2016). This force-mediated activation of thick filaments is thought to allow the non-uniform SLs in muscle fibers to remain stable during isometric contractions, despite differences in actomyosin overlap between sarcomeres of varying lengths (Schappacher-Tilp, 2018). However, it is also postulated that a threshold limit to the SL non-uniformity exists, after which the force-mediated regulation by thick filaments would become ineffective in stabilizing SLs (Schappacher-Tilp, 2018). For example, SLs of isolated myofibrils become highly non-uniform (CV of 25–30%) upon Ca^2+^ activation (Johnston et al., 2019) and may have exceeded this threshold limit of SL non-uniformity, thus leading to unstable SLs and increasing SL non-uniformity over the duration of isometric contractions (Pavlov et al., 2009; Johnston et al., 2019). In the intact muscle, however, the collagenous extracellular matrices, in the form of the endo- and perimysium, provide structural stability to the muscle fibers (Purslow, 1989, 2008; Gillies and Lieber, 2011), thereby limiting the *in situ* SL non-uniformity to CV of < 10% (Figure 4C, see also Moo et al., 2017a; Moo and Herzog, 2018). Therefore, even though the SLs in activated intact muscles are non-uniform, the SLs could stay stable through the force-mediated regulation by thick filaments. Nevertheless, it should be noted that even after taking into account the force-mediated regulating mechanism for SL stabilization, the reduced SL non-uniformity (i.e., decreasing CV, Figure 5) over the contraction time remains an unexplained phenomenon requiring further research to elucidate its mechanism.

When sarcomeres are stretched along the descending limb of the force-length curve (from G2 to G4), the growing relative force contribution from passive elements is accompanied by a decrease in active force due to continuous loss of actomyosin overlap. The interplay of passive and active relative forces likely leads to: (i) a reduction in sarcomere shortening upon activation (Figure 4B), (ii) an overall decrease in SL non-uniformity (Figure 4C), and (iii) a reduced perturbation of SLs upon activation (Figure 4C), at long (G3–G4) compared to short SLs (G1–G2) in TA muscles for fixed-end isometric contractions. The time-dependent decrease of SL non-uniformity also appears to be influenced by the relative contribution of passive and active forces at the different SLs. When the active forces are greater than the passive forces (G1–G3, Figure 6), convergence to SL uniformity over time was achieved by a shortening of the longest sarcomeres (defined by the 95th percentile of SLs), and little or no change in length of the shortest sarcomeres (defined by the 5th percentile of SLs). The opposite occurred when the passive forces exceeded the active forces (G4, Figure 6). These observations suggest that engaging the intracellular passive elements, such as titin molecules, by mechanical stretch, does not only lead to a more uniform SL distribution, but also alter the contraction dynamics of sarcomeres (Horowits and Podolsky, 1987; Powers et al., 2014, 2017).

There are limitations in this study that need to be considered. First, the results presented here were derived from the measurements of sarcomeres in mid-TA, and may not represent sarcomeres from other locations of the muscle, although previous studies suggested that contraction-induced changes of SL distribution are similar between middle and distal TA locations (Moo and Herzog, 2018). Second, the decrease in SL non-uniformity only represents the contraction dynamics of sarcomeres over a 600 ms period. Longer contraction duration may lead to different observations. Third, only muscle fibers in the top 50 μm were investigated. Extrapolation of the results into deeper regions of the muscle should be done with caution (O’Connor et al., 2016).

## Conclusion

Based on the results of this study, we conclude that sarcomeres in the mid-belly of maximally contracting whole muscles constantly re-organize their lengths into a more uniform pattern over time. Furthermore, we conclude that SL non-uniformity and SL dynamics depend in a crucial manner on muscle/sarcomere lengths and appear to be affected by the relative contributions of passive and active forces to the total muscle forces. These results suggest that SL non-uniformity are based on different mechanisms in active and passive muscles, and undergo a perturbation upon activation as they go from one state to the next. The details of the molecular mechanisms accounting for SL non-uniformity in muscles need further elucidation, as do the functional implications of the SL non-uniformity.

## Data Availability Statement

The datasets generated for this study are available on request to the corresponding author.

## Ethics Statement

The animal study was reviewed and approved by University of Calgary’s Life Sciences Animal Research and Ethics Committee.

## Author Contributions

EM and WH contributed to the substantial contributions to the conception or design of the work, or the acquisition, analysis, or interpretation of data for the work, drafting the work or revising it critically for important intellectual content, final approval of the version to be published, and agreement to be accountable for all aspects of the work in ensuring that questions related to the accuracy or integrity of any part of the work are appropriately investigated and resolved.

## Conflict of Interest

The authors declare that the research was conducted in the absence of any commercial or financial relationships that could be construed as a potential conflict of interest.
