# Supplementary material for: Sarcomere Lengths Become More Uniform Over Time in Intact Muscle-Tendon Unit During Isometric Contractions
Source: Front Physiol. 2020 May 12;11:448. doi: 10.3389/fphys.2020.00448 (PMC7235410; doi:10.3389/fphys.2020.00448)
Supplement: Supplementary file 1 [file Table_1.docx]

Supplementary Material

Sarcomere Lengths Become More Uniform Over Time in Intact Muscle-Tendon Unit During Isometric Contractions

**Eng Kuan Moo^1^, Walter Herzog^1, 2*^**

^1^Human Performance Laboratory, Faculty of Kinesiology, University of Calgary, Calgary, Alberta, Canada

^2^Health Centre, Federal University of Santa Catarina, SC, Brazil

*** Correspondence:**Name: Walter Herzog

Address: Human Performance Laboratory, University of Calgary, 2500 University Drive N.W., Calgary, Alberta, T2N 1N4, Canada.

Tel: +1 403 220 8525; E-mail: [wherzog@ucalgary.ca](mailto:wherzog@ucalgary.ca)


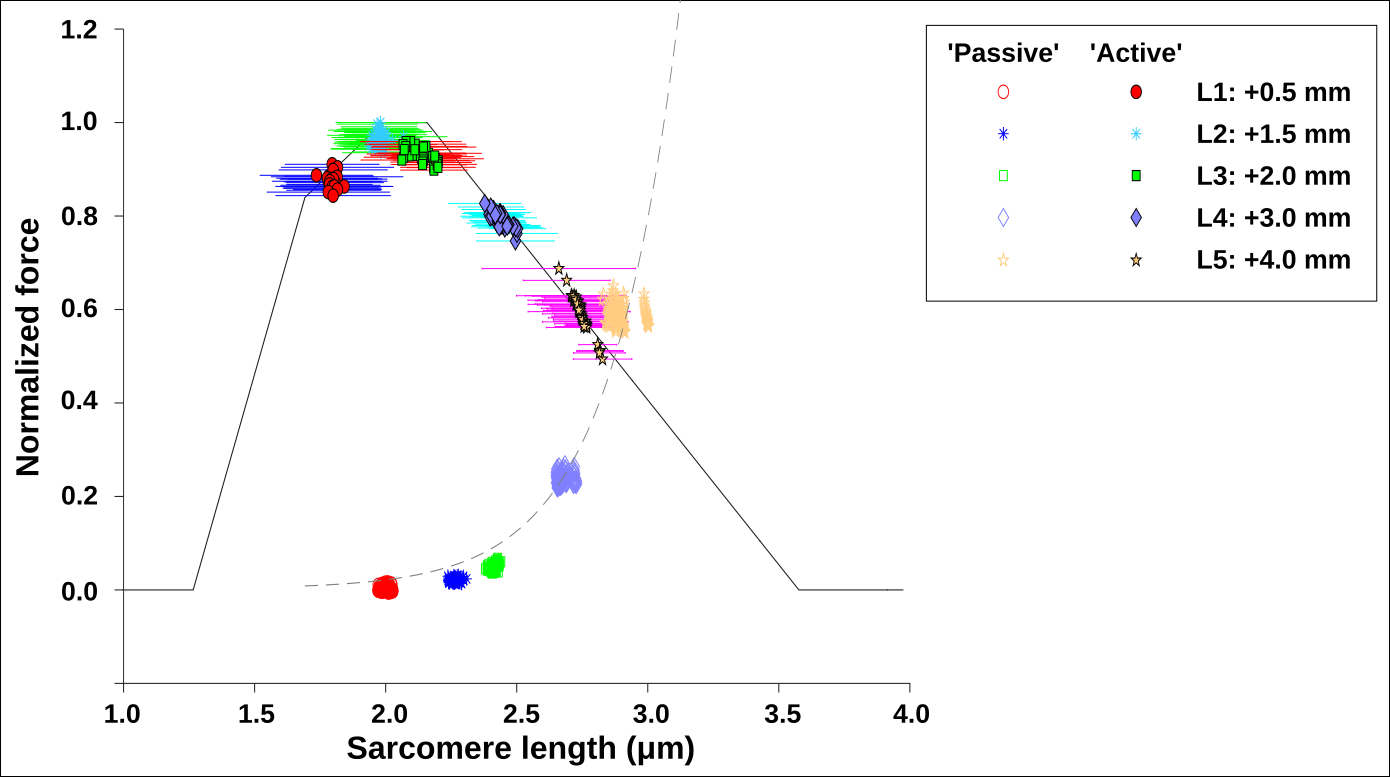


**Fig. S1.** A representative example of a sarcomere FL relationship in the relaxed/passive (markers with coloured outline) or activated (markers with coloured face and black outline) condition for animal #5. Sarcomere lengths were measured in individual image bands, and temporally linked to the corresponding force traces. The horizontal bars associated with the active FL data represent the standard deviation of the sarcomere lengths in individual image bands. This information is excluded from the passive FL data for clarity purposes. The inverted U-shaped curve (solid black line) is the theoretical FL curve, whereas the exponential curve (dotted grey line) represents the curve best-fitted to passive FL data.


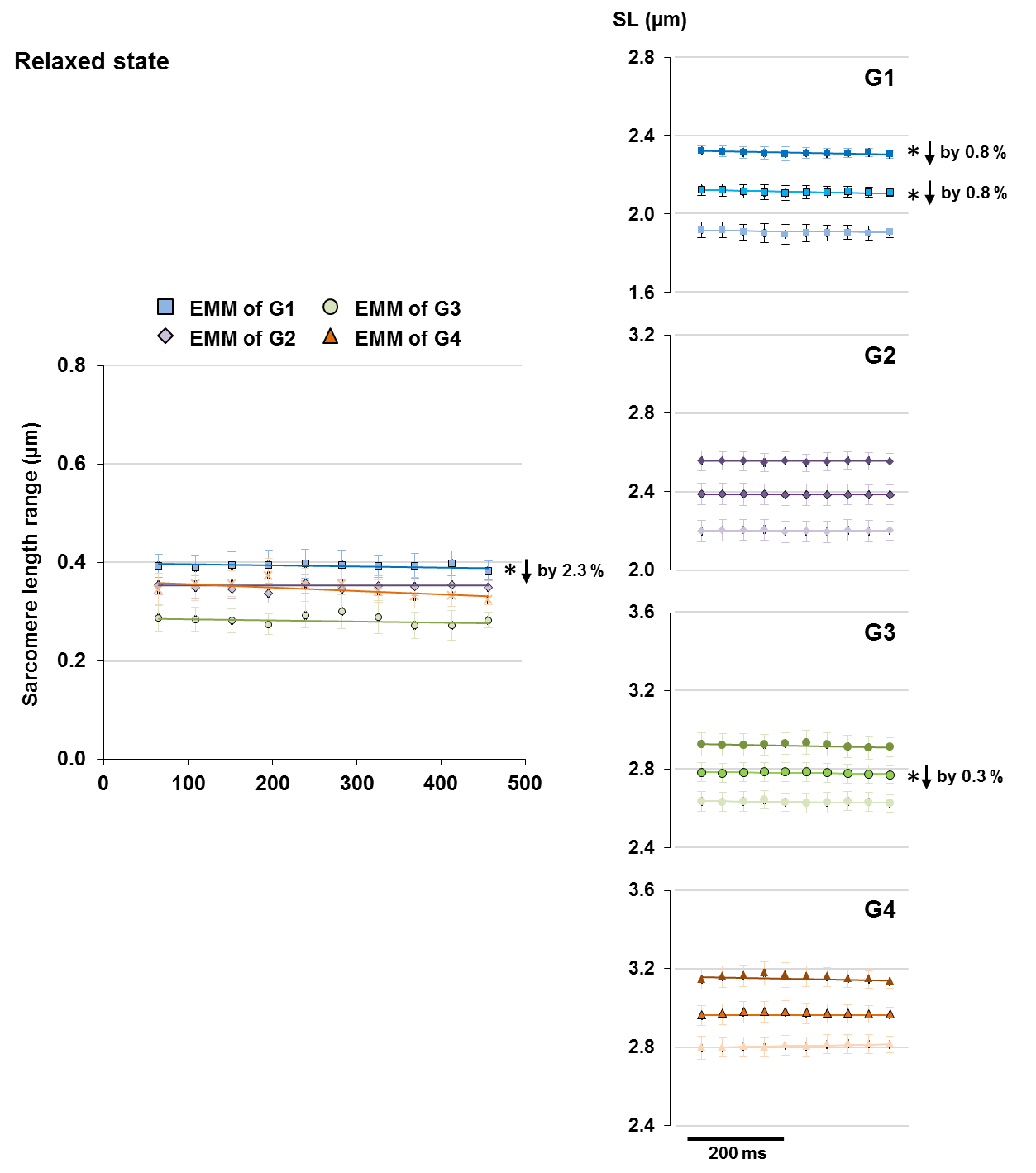


**Fig. S2.** Length range of sarcomeres observed in individual image bands of relaxed muscles (left column), and the corresponding changes of 95^th^ percentile (top curve), average (middle curve), and 5^th^ percentile (bottom) of SLs in each of the SL group (G1 – 4, right column). * indicates significant relationship between time and either SL range, 5^th^ percentile, average or 95^th^ percentile of the SL (p < 0.01). EMM: estimated marginal mean.

Table S1: Summary of the number of analyzed images and sarcomeres for each MTU length group (L1 – 5). Note that the analyzed images contain repeat measurements of the same group of sarcomeres in single trials, but this is accounted for in the reported number of analyzed sarcomeres.

|  |  | **Number of analyzed images** | | **Number of analyzed sarcomeres** | |
| --- | --- | --- | --- | --- | --- |
| **Group** | **Stretch (mm)** | **Relaxed** | **Activated** | **Relaxed** | **Activated** |
| L1 | 0.5 | 602 | 219 | 1904 | 1534 |
| L2 | 1.5 | 560 | 258 | 1812 | 2048 |
| L3 | 2.0 | 568 | 264 | 1658 | 2042 |
| L4 | 3.0 | 549 | 300 | 1658 | 2036 |
| L5 | 4.0 | 545 | 323 | 1482 | 1960 |

Table S2: Summary of the number of analyzed images and sarcomeres for each sarcomere length group (G1 – 4) after group re-assignment. Note that the analyzed images contain repeat measurement of the same group of sarcomeres in single trials, but this is accounted for in the reported number of analyzed sarcomeres (see Methods for more details).

|  | **Average SLs (µm)** | **Number of analyzed images** | | **Number of analyzed sarcomeres** | |
| --- | --- | --- | --- | --- | --- |
| **Group** |  | **Relaxed** | **Activated** | **Relaxed** | **Activated** |
| G1 | 1.80 – 2.00 | 727 | 327 | 2364 | 2290 |
| G2 | 2.15 – 2.30 | 932 | 393 | 2738 | 3076 |
| G3 | 2.55 – 2.75 | 534 | 295 | 1642 | 2012 |
| G4 | 2.90 – 3.15 | 423 | 254 | 1146 | 1522 |

Table S3: Maximal apparent passive (relaxed) and active (activated) forces (mean ± S.E.) for each sarcomere length group (G1 – 4) to which the data presented in Fig. 7 were normalized.

| **Group** | **Number of trials** | | **Maximal apparent force within individual trials (mN)** | |
| --- | --- | --- | --- | --- |
|  | **Relaxed** | **Activated** | **Relaxed/Passive** | **Activated** |
| G1 | 40 | 48 | 290 ± 20 | 2164 ± 28 |
| G2 | 50 | 54 | 298 ± 18 | 2249 ± 42 |
| G3 | 25 | 34 | 866 ± 79 | 1195 ± 80 |
| G4 | 25 | 28 | 2028 ± 86 | 734 ± 37 |

**Some remarks on reproducibility of SL non-uniformity**

Reproducibility of SL distribution between trials was not specifically tested here due to the current study design. Also, one of the reasons for this was because we were not sure what it would mean if the SL distributions were not reproducible. Would that mean that the experiment was performed badly, or that SL distributions in a muscle are random and different every time a muscle changes length or produces force, and thus cannot be predicted?

The question of whether SL non-uniformity is random or repeatable needs to be addressed on a smaller structural level first where each sarcomere can be uniquely identified and its length measured for different, repeated contractile conditions. We are presently doing such experiment with single myofibril preparations containing approximately 20 sarcomeres in series. Pilot results seem to suggest that each time a myofibril is activated at a given length and reaches a given force, the SL distribution (in fact the length of individual sarcomeres) is the same. When the myofibril is deactivated, the individual SLs change dramatically, but appear to go back to the state/lengths they were before. These pilot results suggest that (i) SLs are not random, but are governed by some internal sarcomeric structure, and that (ii) the elements producing SL non-uniformity are distinctly different for the active and the passive muscle. However, this study could not have been done on an entire muscle as tested here because it was not possible to uniquely identify a given sarcomere each time in the passive and active states of the TA muscle.
